# Supplementary material for: Probing aggrephagy using chemically-induced protein aggregates
Source: Nat Commun. 2018 Oct 12;9:4245. doi: 10.1038/s41467-018-06674-4 (PMC6185936; doi:10.1038/s41467-018-06674-4)
Supplement: Supplementary file 1 — Supplementary Information [file 41467_2018_6674_MOESM1_ESM.pdf]

Supplementary Information

Probing aggrephagy using chemically-induced protein aggregates

Janssen *et al.*

Supplementary Figure 1

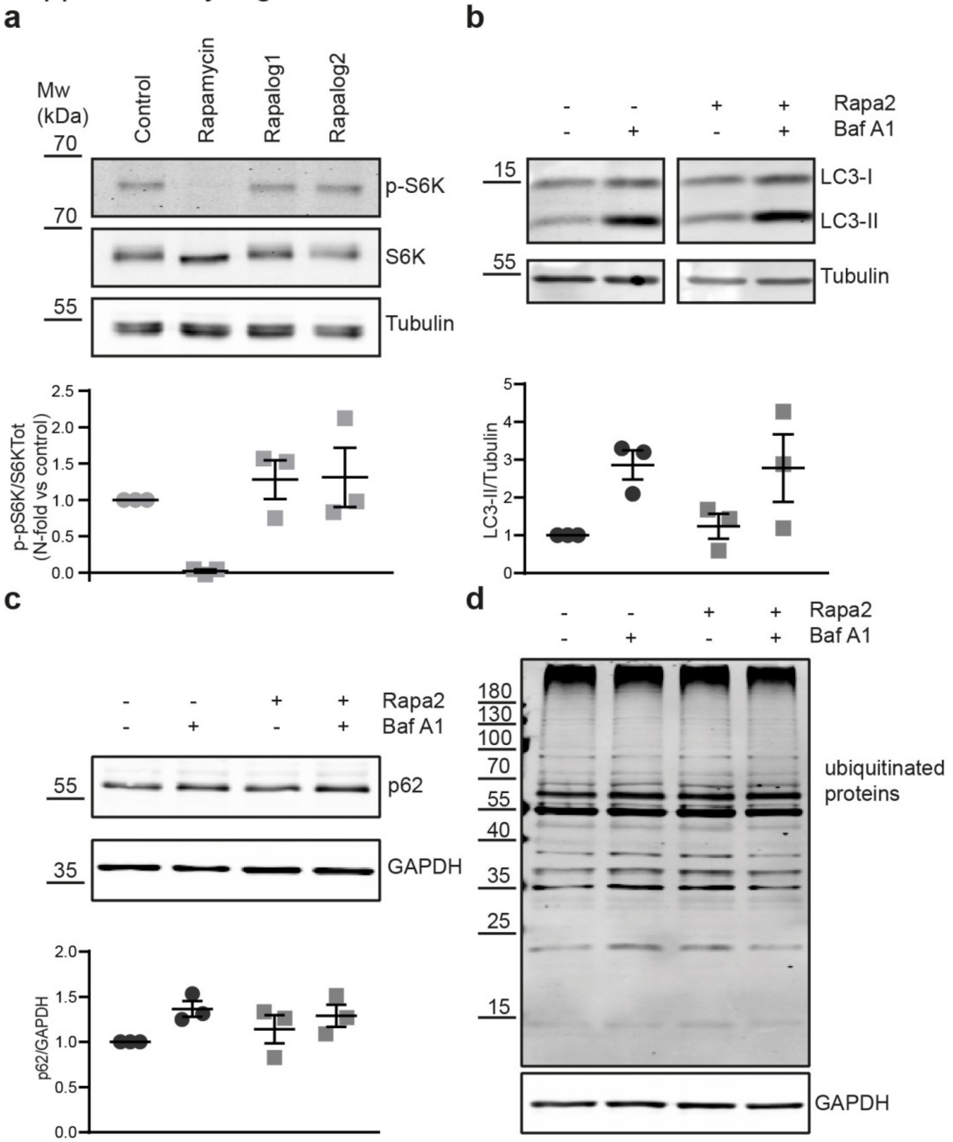

Supplementary Figure 1: Rapalog2 alone does not affect autophagy

(a) pS6K blot for mTORC1 activity. Phospho-S6K, S6K and α-tubulin immunoblot of whole cell lysates of Hela cells treated with rapamycin, rapalog1 and rapalog2. Representative blot of 3 independent experiments. Data are expressed as mean ± s.e.m, individual data points are shown (n=3). (b) LC3 immunoblot of cells treated with rapalog2 and/or Bafilomycin A1. Representative blot of 3 independent experiments. Data are expressed as mean ± s.e.m. (n=3). (c) p62 immunoblot of cells treated with rapalog2 and/or Bafilomycin A1. Representative blot of 3 independent experiments. Data are expressed as mean ± s.e.m. (n=3). (d) p62 immunoblot of cells treated with rapalog2 and/or Bafilomycin A1. Representative blot of 3 independent experiments.

Supplementary Figure 2

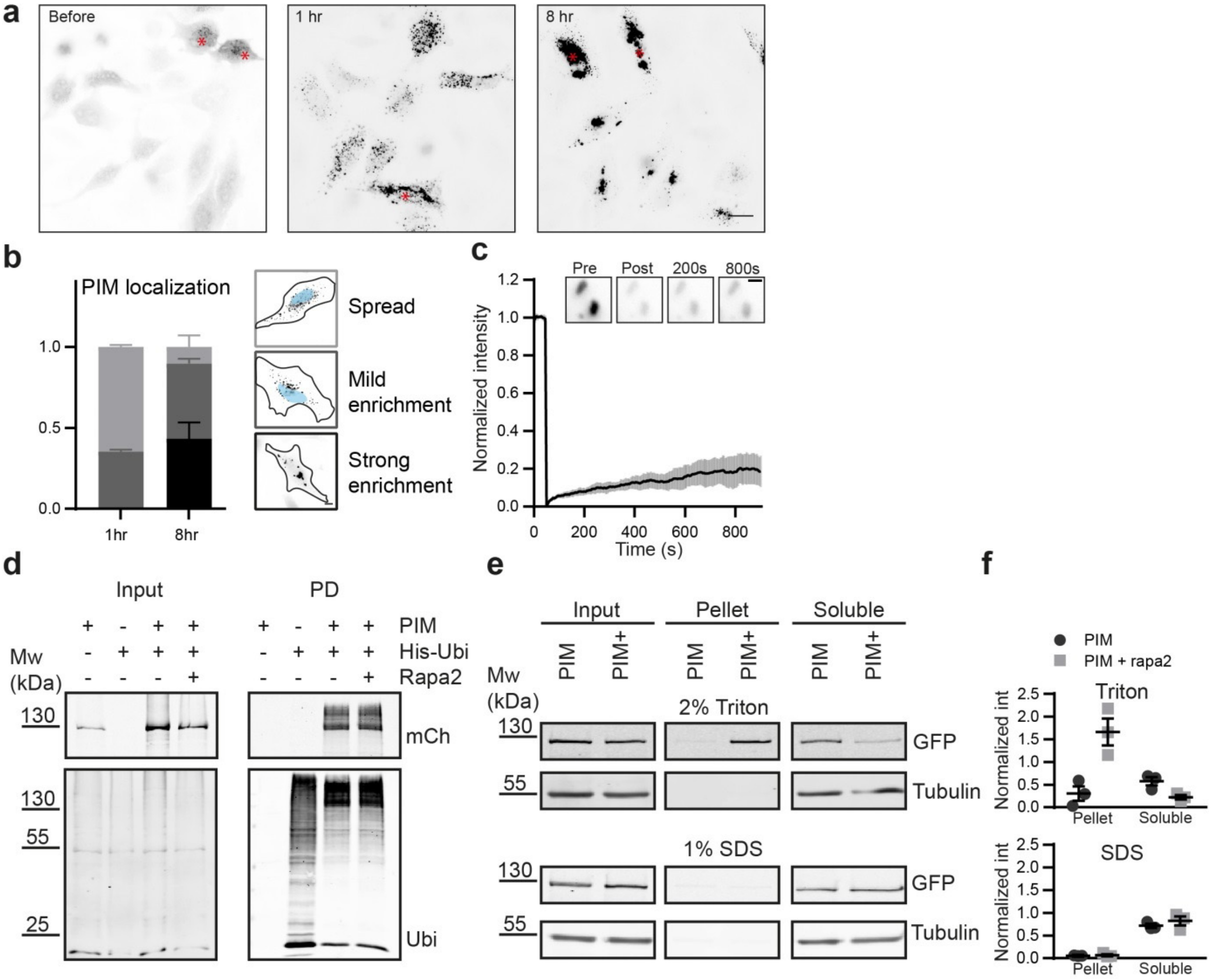

Supplementary Figure 2: PIM cluster characterization

(a) Images of HeLa cells transiently expressing the PIM construct before, 1 or 8 h after rapalog2 addition. Asterisks indicate cells that were excluded from analysis due to high aggregation levels. (b) Quantification of manually scored PIM localization using mCherry channel in fixed cells at different time points. Categories used were spread localization (light grey), mild (dark grey) or strong (black) perinuclear accumulation. Quantification of 16-27 cells per experiment per time point. Data represents mean ± s.e.m of 3 independent experiments. (c) FRAP recovery curves of clusters 4 h after aggregate formation. Mean ± s.e.m (n=7). Inserts show example images of FRAP measurements. (d) PIM construct was co-transfected with His-Ubi into HEK293 cells. Cells were lysed under denaturing conditions 4 h after aggregate formation, after which Ni-NTA pull-downs assays were performed. A representative image illustrating 2 experiments is shown. (e) Triton X-100 and SDS soluble and insoluble fractions obtained without rapalog2 induction (PIM) and 4 h after rapalog2 induction (PIM+). A representative image illustrating 3 independent experiments is shown for Triton X-100 and SDS fractionation. (f)

Quantification of data in (e). Data are expressed as mean ± s.e.m. (n=3). Scale bars, 20μm (a), 10 μm (b) and 1 μm (c).

Supplementary Figure 3

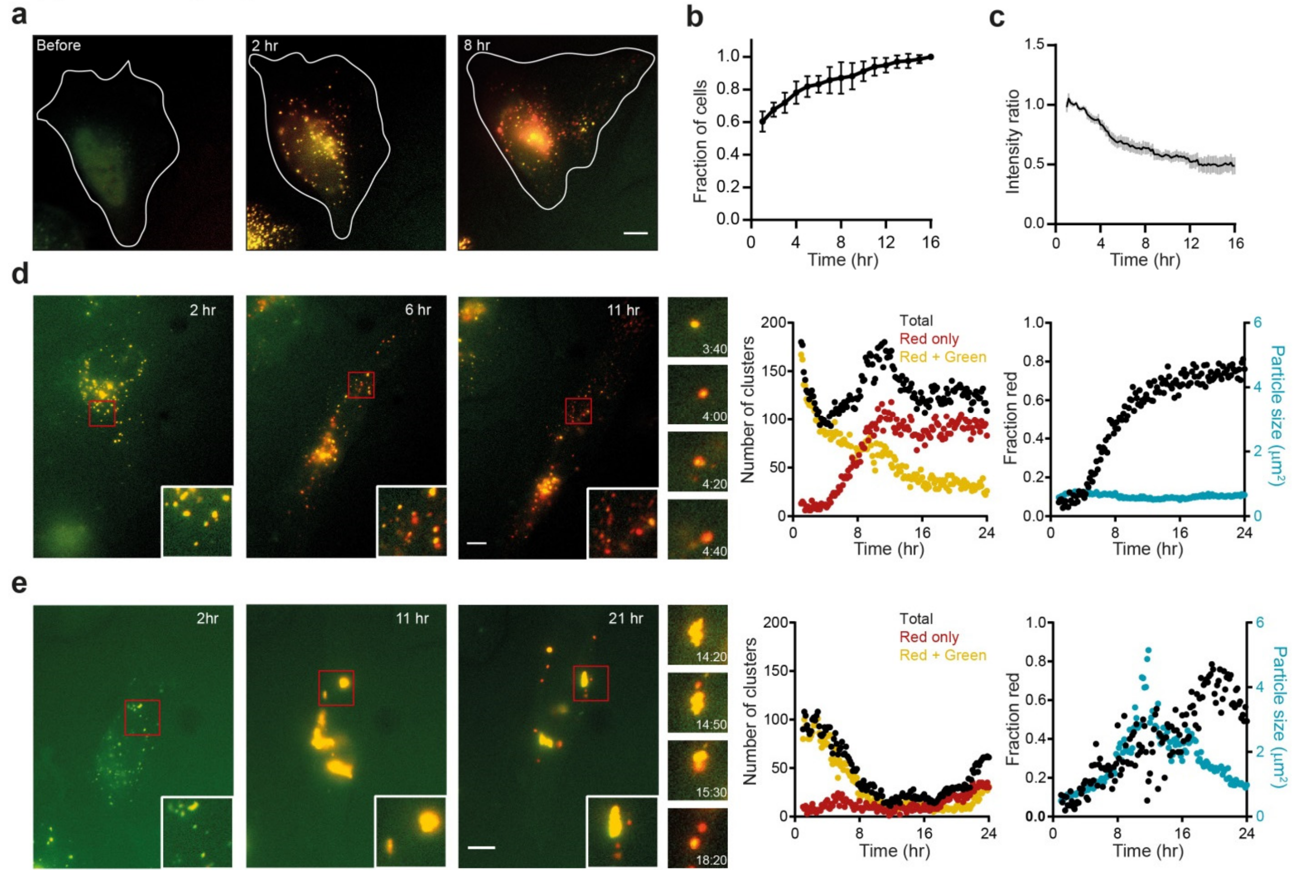

Supplementary Figure 3: Live-cell cluster degradation in individual cells

(a) Full-sized merged images of the cell in Fig. 1b. (b) Fraction of cells with PIM particles as a function of time after rapalog2 addition during 16 h live cell imaging. Data represents mean ± s.e.m. of 3 independent experiments. (c) Total GFP/mCherry intensity ratio of all detected clusters per cell during 16 h live cell imaging. Alternative analysis of data in Fig1f-g. Data are mean± s.e.m. n=11 cells from 3 independent experiments. (d-e) Analysis of individual cells showing two representative responses to cluster induction, i.e. direct clearance of smaller aggregates (d) or merging followed by clearance (e). Left graph, number of total (black), red only (red) and red and green (yellow) clusters over time. Right graph, average detected particle size (blue) and the fraction of red clusters (black) over time. Time indicates time after rapalog2 addition. Small panels show degradation of smaller clusters (d) or larger merged clusters (e) over time. Scale bars, 10 µm (a,d,e).

Supplementary Figure 4

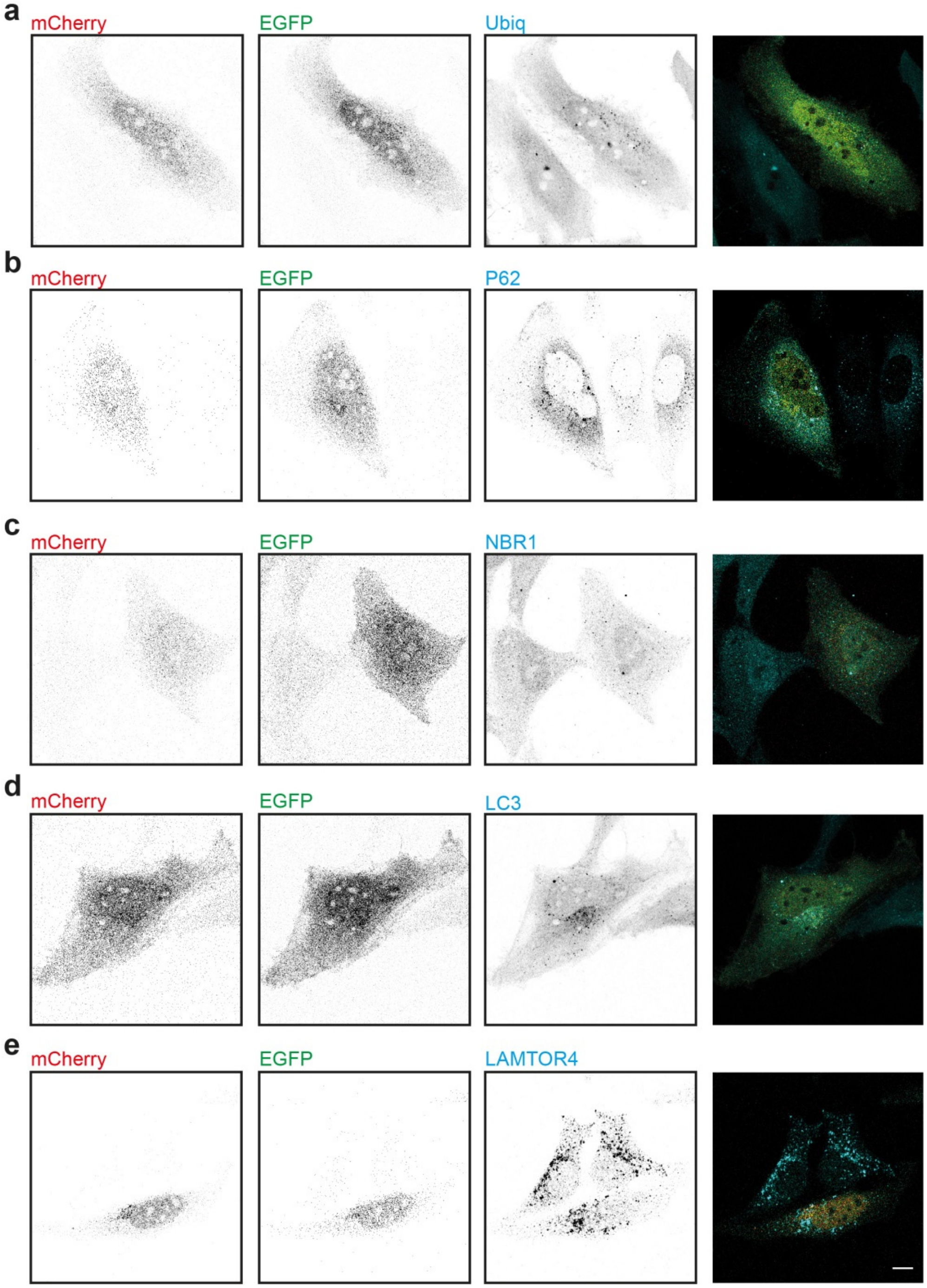

Supplementary Figure 4: Autophagic machinery markers in PIM-construct expressing cells

Immunofluorescence images of representative PIM-construct expressing cells without rapalog2 addition. mCherry (Red), EGFP (green) and endogenous staining (cyan) of ubiquitin (a), p62 (b), NBR1 (c), LC3 (d) and LAMTOR4 (e) shown in inverted contrast. Scale bar, 10 µm. This data correspond to Fig 2.

Supplementary Figure 5

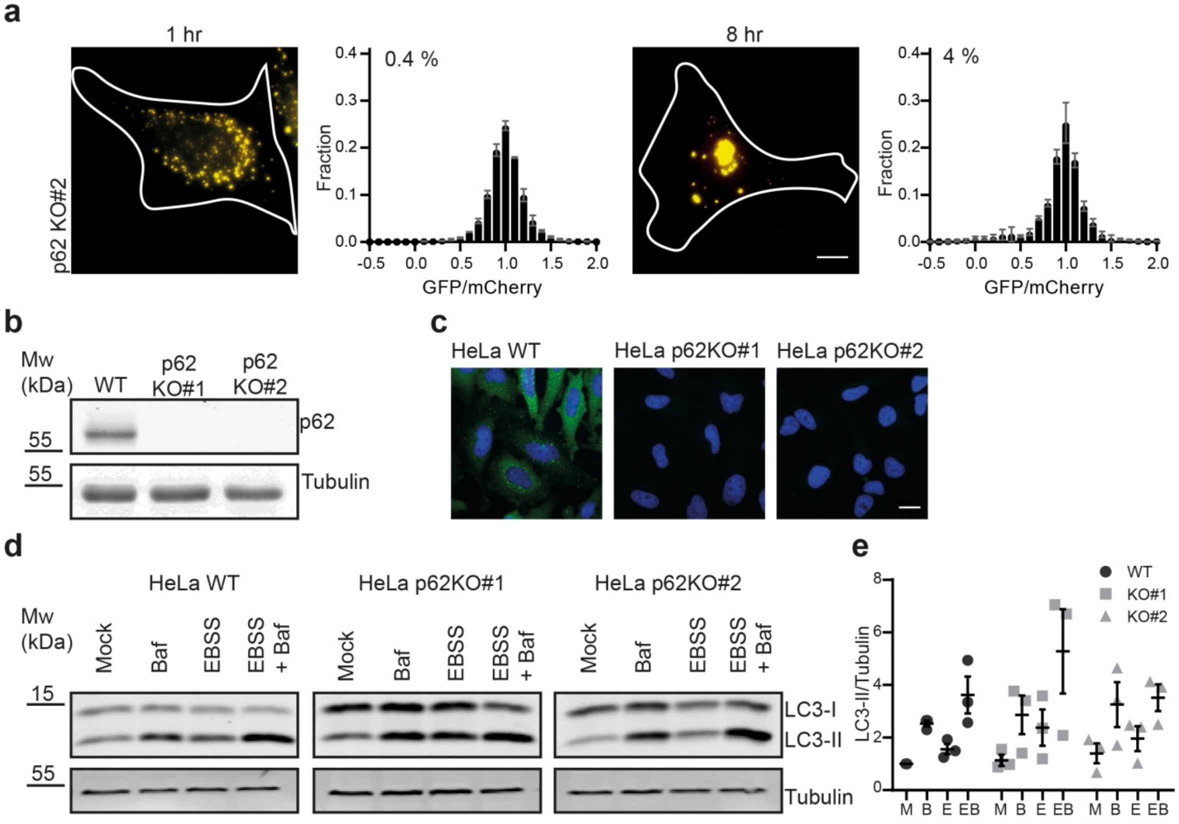

Supplementary Figure 5: CRISPR-Cas9 mediated KO of p62 in HeLa cells.

(a) Distribution of normalized EGFP/mCherry ratios of clusters at different time points after cluster formation in the HeLa p62KO line #2. Each histogram represents over 3000 clusters. Mean ± s.e.m from 3 independent experiments with in total 40-60 cells per condition. Percentage of red clusters is indicated as the average fraction of clusters with a EGFP/mCherry ratio below 0.3 of 3 independent experiments. (b) p62 and Tubulin immunoblot of whole-cell lysates from HeLa WT and HeLa p62 KO cells. Representative blots are shown (n=3) (c) Representative images of endogenous p62 in WT and p62 KO HeLa cell lines. (d) Western blot analysis of LC3-II production in Hela WT and p62 KO lines. Cells were treated with Bafilomycin A1 under control and starvation (EBSS) conditions. Representative blots are shown (n=3). (e) LC3-II production of Mock (M), Bafilomycin (B), EBSS (E) and EBSS and Bafilomycin (EB) treated samples of HeLa WT (WT, black bars), HeLa p62KO#1 (KO#1, light grey bars) and HeLa p62KO#2 (KO#2, dark grey bars) were quantified. Data are expressed as mean ± s.e.m. (n=3). Scale bars, 10 μm (a) en 20 μm (c)

Supplementary Figure 6

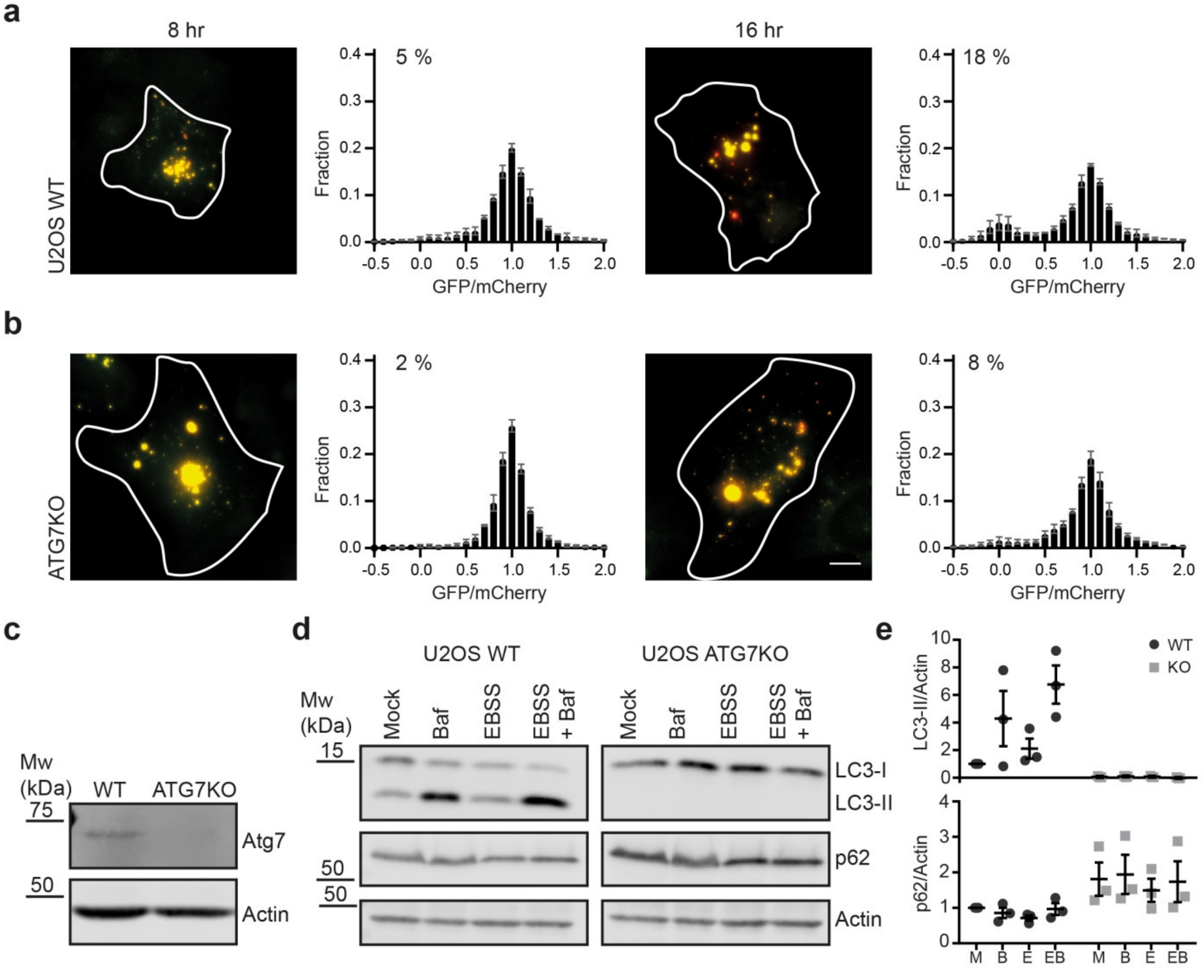

Supplementary Figure 6: Aggregate clearance in U2OSAtg7KO cells

(a,b) Distribution of normalized EGFP/mCherry ratios of clusters at 8 and 16 h after cluster formation in U2OS WT cells (a) and U2OSAtg7KO cells (b) Each histogram represents over 5000 clusters. Mean ± s.e.m from 3 independent experiments with in total 40-60 cells per condition. Percentage of red clusters is indicated as the average fraction of clusters with a EGFP/mCherry ratio below 0.3 of 3 independent experiments. (c) Atg7 and actin immunoblot of whole-cell lysates from U2OS WT and Atg7KO cells. (d) LC3, p62 and actin immunoblot of whole-cell lysates from U2OS WT and Atg7KO cells of cells treated with Bafilomycin A1 under control and starvation (EBSS) conditions. (e) LC3-II production of Mock (M), Bafilomycin (B), EBSS (E) and EBSS and Bafilomycin (EB) treated samples of U2OS WT (WT, black bars) and U2OSAtg7KO (KO, grey bars) were quantified. Data are expressed as mean ± s.e.m. (n=3). Scale bar 10 μm.

Supplementary Figure 7

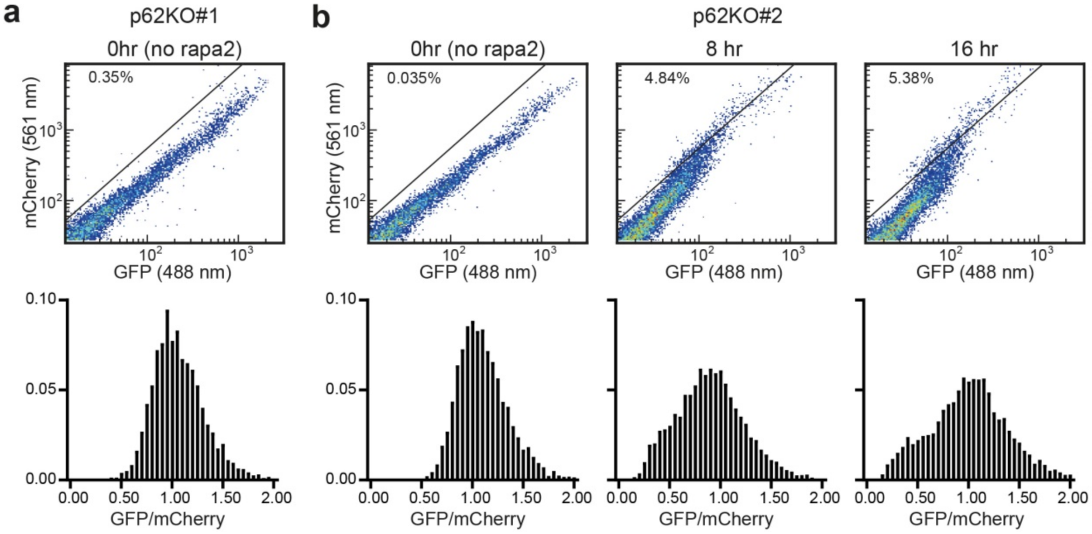

Supplementary Figure 7: FACS analysis of HeLa p62KO#2

Cells were treated with rapalog2 and analyzed by FACS for a shift in GFP/mCherry ratio. Scatter plots and matching histograms of GFP/mCherry ratio are shown. (a) Representative data for HeLa p62KO#1 line before rapalog2 addition. (b) Representative data for HeLa p62KO#2 cells before, 8 and 16 h after rapalog2 addition. Representative plots from 3 experiments are shown.

Supplementary Figure 8

Supplementary figure 1a

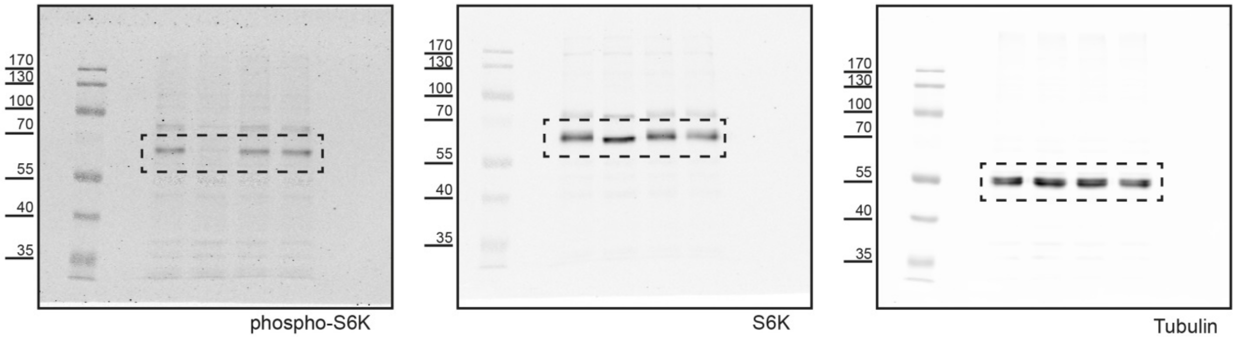

Supplementary figure 1b

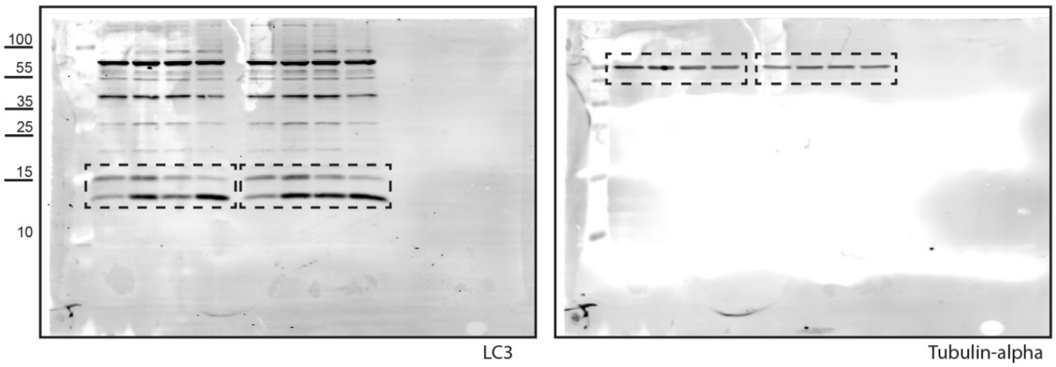

Supplementary figure 1c

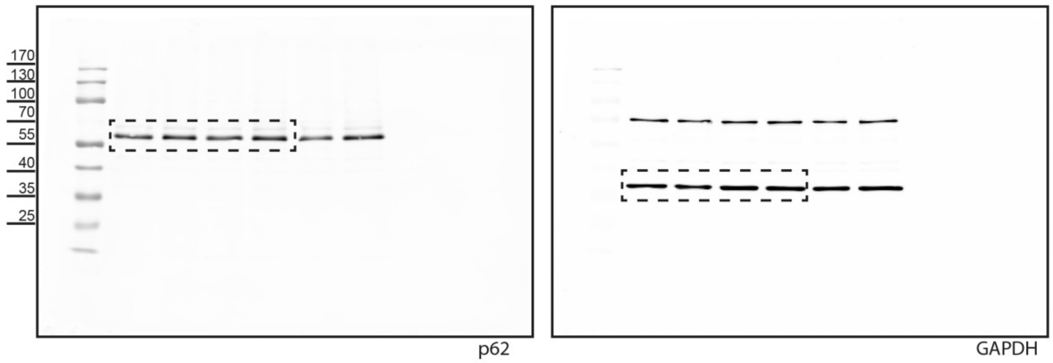

Supplementary figure 1d

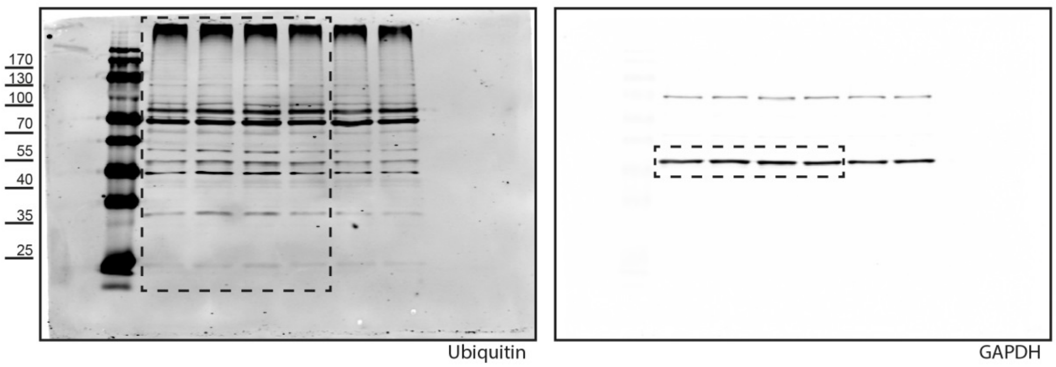

Supplementary figure 2d

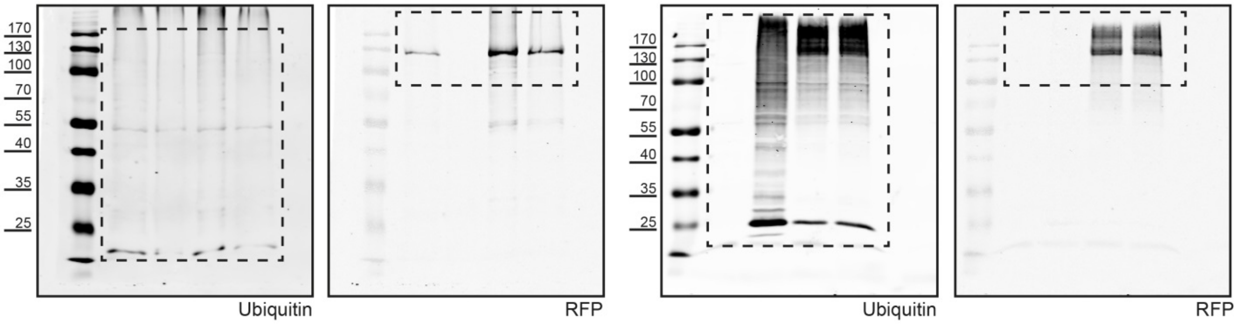

Supplementary figure 2e

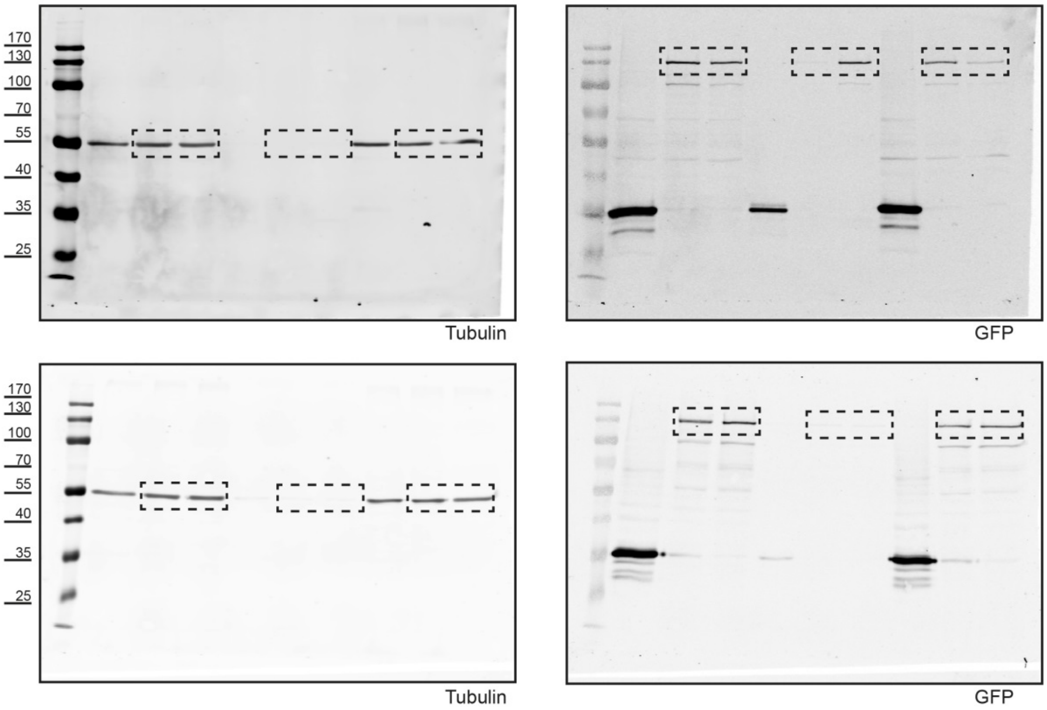

Supplementary figure 5b

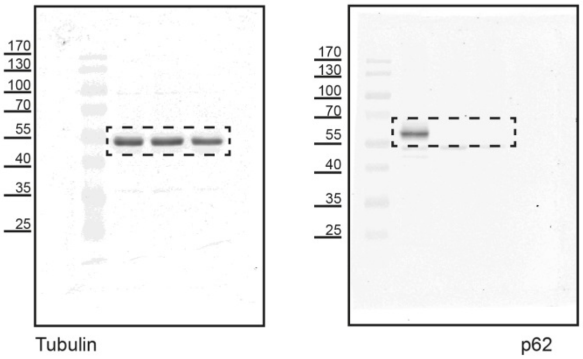

Supplementary figure 5d

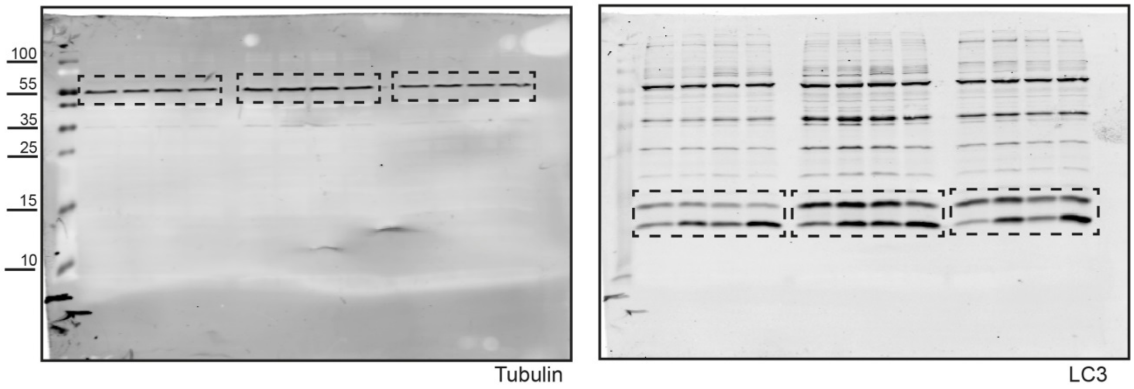

Supplementary figure 6c

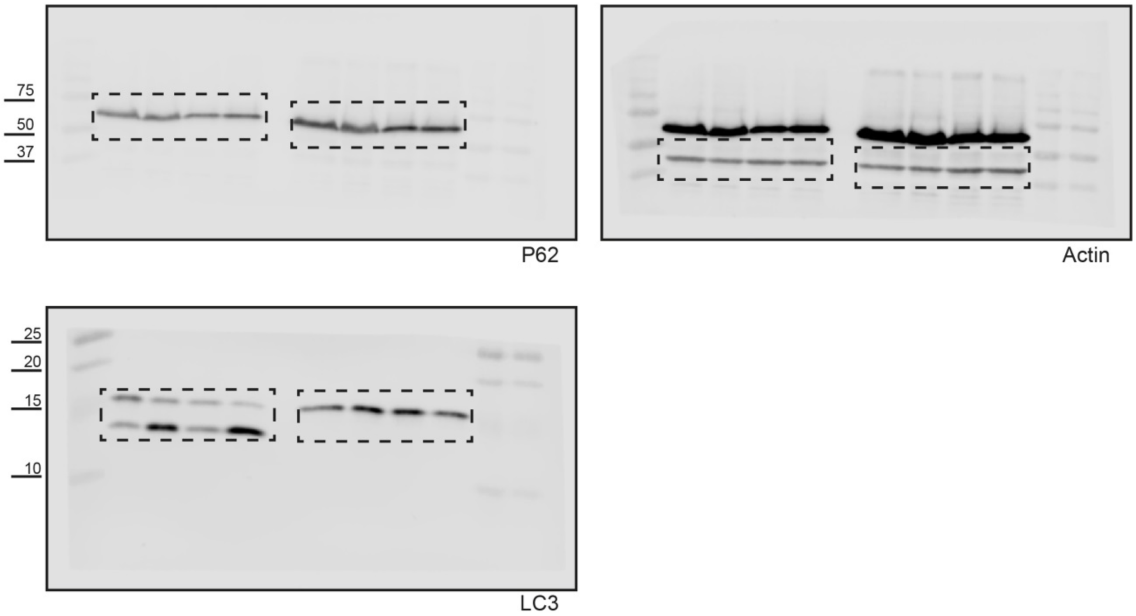

Supplementary figure 6d

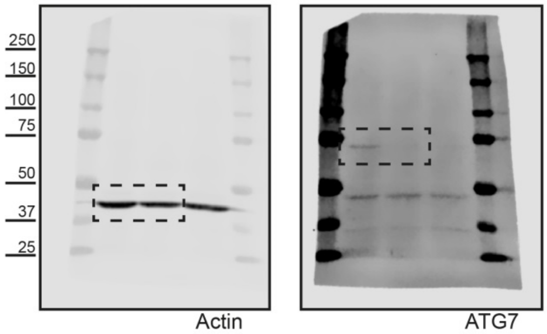

Supplementary Figure 8: Full scans of Western blot images.

Supplementary Figure 9

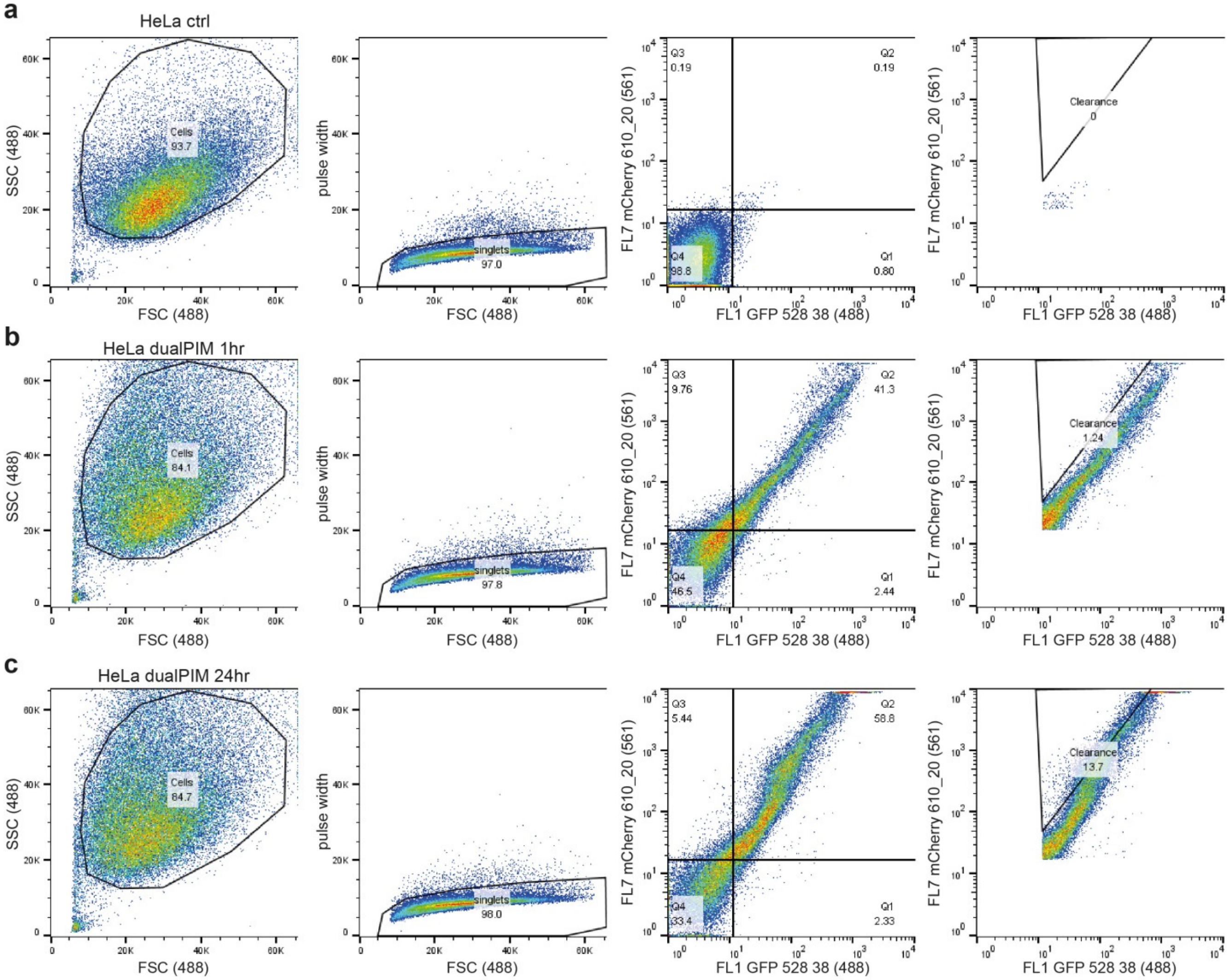

Supplementary Figure 9: Gating strategy for FACS experiments

Gating strategy used for all FACS analysis. Left plots show ungated plots with the gate "cells" used to gate out cell debris (first column). Cells were then gated based on pulse width to gate out cell clumps and obtain single cell population (second column). Subsequently, transfected cells were distinguished on the basis of GFP and mCherry fluorescence using non-transfected cells to determine the gates (a, column 3). Finally, to determine the cells that showed aggregate clearance, gates were set on basis of cells expressing the dualPIM construct 1 hr after aggregate formation (b, column 4). The gating strategy was shown for untransfected HeLa (a), HeLa expressing the dualPIM construct 1hr after aggregate induction (b) and HeLa expressing the dualPIM construct 24hr after aggregate induction (c).
