# Supplementary material for: Probing aggrephagy using chemically-induced protein aggregates
Source: Nat Commun. 2018 Oct 12;9:4245. doi: 10.1038/s41467-018-06674-4 (PMC6185936; doi:10.1038/s41467-018-06674-4)
Supplement: Supplementary file 3 — Description of Additional Supplementary Files [file 41467_2018_6674_MOESM3_ESM.pdf]

Description of Additional Supplementary Files

File Name: Supplementary Movie 1
Description: This movie complements Figure 1b. HeLa cell expressing PIM construct showing cluster formation and degradation. Left panel shows inverted contrast gray scale images of mCherry channel while right panel shows merged channel of mCherry (red) and EGFP (green) channel. Time is indicated in hours. 3 min between frames.

File Name: Supplementary Movie 2
Description: This movie complements Figure 1b. HeLa cell expressing PIM construct showing cluster formation and degradation. Left panel shows inverted contrast gray scale images of mCherry channel while right panel shows merged channel of mCherry (red) and EGFP (green) channel. Time is indicated in hours. 3 min between frames.
