# Supplementary material for: Probing aggrephagy using chemically-induced protein aggregates
Source: Nat Commun. 2018 Oct 12;9:4245. doi: 10.1038/s41467-018-06674-4 (PMC6185936; doi:10.1038/s41467-018-06674-4)
Supplement: Supplementary file 6 — Reporting Summary [file 41467_2018_6674_MOESM6_ESM.pdf]

nature research

Corresponding author(s): Lukas C. Kapitein



# Reporting Summary

Nature Research wishes to improve the reproducibility of the work that we publish. This form provides structure for consistency and transparency in reporting. For further information on Nature Research policies, see Authors & Referees and the Editorial Policy Checklist.

## Statistical parameters

When statistical analyses are reported, confirm that the following items are present in the relevant location (e.g. figure legend, table legend, main text, or Methods section).

| n/a | Confirmed | |
|---|---|---|
| ☐ | ☒ | The exact sample size (*n*) for each experimental group/condition, given as a discrete number and unit of measurement |
| ☐ | ☒ | An indication of whether measurements were taken from distinct samples or whether the same sample was measured repeatedly |
| ☐ | ☒ | The statistical test(s) used AND whether they are one- or two-sided<br>*Only common tests should be described solely by name; describe more complex techniques in the Methods section.* |
| ☒ | ☐ | A description of all covariates tested |
| ☐ | ☒ | A description of any assumptions or corrections, such as tests of normality and adjustment for multiple comparisons |
| ☐ | ☒ | A full description of the statistics including central tendency (e.g. means) or other basic estimates (e.g. regression coefficient) AND variation (e.g. standard deviation) or associated estimates of uncertainty (e.g. confidence intervals) |
| ☐ | ☒ | For null hypothesis testing, the test statistic (e.g. *F*, *t*, *r*) with confidence intervals, effect sizes, degrees of freedom and *P* value noted<br>*Give P values as exact values whenever suitable.* |
| ☒ | ☐ | For Bayesian analysis, information on the choice of priors and Markov chain Monte Carlo settings |
| ☒ | ☐ | For hierarchical and complex designs, identification of the appropriate level for tests and full reporting of outcomes |
| ☒ | ☐ | Estimates of effect sizes (e.g. Cohen's *d*, Pearson's *r*), indicating how they were calculated |
| ☐ | ☒ | Clearly defined error bars<br>*State explicitly what error bars represent (e.g. SD, SE, CI)* |

*Our web collection on statistics for biologists may be useful.*

## Software and code

Policy information about availability of computer code

| | |
|---|---|
| Data collection | Micromanager (ImageJ), MetaMorph and LAS-X software were used for collection of images. Image Studio software on Odyssey scanner for western blots. |
| Data analysis | ComDet v.0.3.7 plugin for ImageJ was used for detection and analysis of aggregate intensities. The code is available online (https://github.com/ekatrukha/ComDet).Western Blot analysis was performed using ImageJ. Furthermore data analysis was performed using Microsoft Excel and Graphpad PRISM7 software. |

For manuscripts utilizing custom algorithms or software that are central to the research but not yet described in published literature, software must be made available to editors/reviewers upon request. We strongly encourage code deposition in a community repository (e.g. GitHub). See the Nature Research guidelines for submitting code & software for further information.

## Data

Policy information about availability of data

All manuscripts must include a data availability statement. This statement should provide the following information, where applicable:

- Accession codes, unique identifiers, or web links for publicly available datasets
- A list of figures that have associated raw data
- A description of any restrictions on data availability

All relevant data are available from the authors.

# Field-specific reporting

Please select the best fit for your research. If you are not sure, read the appropriate sections before making your selection.

☒ Life sciences ☐ Behavioural & social sciences ☐ Ecological, evolutionary & environmental sciences

For a reference copy of the document with all sections, see nature.com/authors/policies/ReportingSummary-flat.pdf

# Life sciences study design

All studies must disclose on these points even when the disclosure is negative.

| | |
|---|---|
| Sample size | Sample size was not predetermined. Sample sizes were chosen based on type of experiment, and whether the chosen sample size gave reproducibility of the results over multiple independent experiments. E.g: for fixed cell analysis 15-20 cells per condition were analyzed and this was repeated 3 times. When data were consistent over 3 independent experiments we concluded sample size to be sufficient. |
| Data exclusions | For analysis of live cell cluster clearance, only cells that showed at least some yellow to red conversion and could be followed for at least 16 h were included. Cells that showed late aggregation (i.e. after several hours), died or divided during the imaging session or that at some point lost focus were discarded. In addition, cells in which all PIMs converged to one single cluster were not included. For analysis of fixed samples, cells that were not entirely in field of view were discarded from analysis. Also cells with high aggregate levels resulting in no visible individual aggregates were discarded. |
| Replication | All experiments were replicated at least once, most were performed 3 times independently. All experiments showed reproducibility and no experiments failed to be reproduced. |
| Randomization | Not applicable |
| Blinding | Investigators were not blinded. |

# Reporting for specific materials, systems and methods

### Materials & experimental systems

| n/a | Involved in the study |
|---|---|
| ☒ | ☐ Unique biological materials |
| ☐ | ☒ Antibodies |
| ☐ | ☒ Eukaryotic cell lines |
| ☒ | ☐ Palaeontology |
| ☒ | ☐ Animals and other organisms |
| ☒ | ☐ Human research participants |

### Methods

| n/a | Involved in the study |
|---|---|
| ☒ | ☐ ChIP-seq |
| ☐ | ☒ Flow cytometry |
| ☒ | ☐ MRI-based neuroimaging |

## Antibodies

| | |
|---|---|
| Antibodies used | Mouse anti-p62 (Abnova, #H00008878-M01 2C11, 1/2000 ), rabbit anti-ATG7 (Cell Signaling Technology, #2631S, 1/1000), mouse anti-actin (Merck, #MAB1501, 1/5000), rabbit anti-phospho-S6K(Thr389) (CST, #9205,CST1/5000), rabbit anti-S6K (CST, 49D7#2708, 1/5000, CST), mouse anti-alpha-tubulin- (B-5-1-2, Sigma, #T5168, 1/20000). mouse anti-p62 (Abcam, #ab56416, 1/2000 ), mouse anti-ubiquitin (Enzo, #BML-PW8810 FK2, 1/2000), rabbit anti-GAPDH (Sigma, #G9545, 1/5000), rabbit anti-LC3 (Novus Biologicals, #NB600-1384, 1/1000), rabbit anti-LC3 (MBL, #PM036, 1/200), mouse anti-p62 (Abnova, #H00008878-M012C11, 1/500), rabbit anti-NBR1 (Novus, #NBP1-71703, 1/500), rabbit anti-LAMTOR4 (CST, #12284SD6A4V, 1/500), rabbit anti-GFP (Abcam, ab290, 1/5000), rabbit anti-RFP (Rockland, Cat. No #600-401-379, 1/2000). |

Validation

All antibodies used in this study are widely used and validated antibodies in the field. Numerous references and validation can be found on the suppliers websites.

# Eukaryotic cell lines

Policy information about cell lines

Cell line source(s)

HeLa and HEK293T cells were purchased from ATCC, U2OS cells were a gift from G. Strous, University Medical Center Utrecht, the Netherlands.

Authentication

None of the cell lines used were authenticated.

Mycoplasma contamination

All cell lines regularly tested negative for mycoplasma contamination

Commonly misidentified lines
(See ICLAC register)

-

# Flow Cytometry

## Plots

Confirm that:

☒ The axis labels state the marker and fluorochrome used (e.g. CD4-FITC).

☒ The axis scales are clearly visible. Include numbers along axes only for bottom left plot of group (a 'group' is an analysis of identical markers).

☒ All plots are contour plots with outliers or pseudocolor plots.

☒ A numerical value for number of cells or percentage (with statistics) is provided.

## Methodology

Sample preparation

HeLa cells (ATCC) were transfected with the DualcolorPIM construct 2 days before analysis. 1 day before analysis treatments were started with rapalog to induce aggregate formation. For analysis, cells were trypsinized and redissolved in PBS.

Instrument

BD-Influx cell sorter (San Jose, CA)

Software

Spigot 6.1.9 software was used to collect data, FlowJo V10 was used for analysis

Cell population abundance

All cells which were transfected and showed EGFP and mCherry fluorescence were analyzed. Depending on the replicate this was ~20-50% of cells analyzed.

Gating strategy

Cells were first gated based on FSC/SSC using the 488 laser as exitation to exclude cell debris. Subsequently single cells were gated using the pulse width. The single cell population was subsequently gated for GFP and mCherry positive cells using HeLa untransfected cells as a negative control to set the gates. Finally of these positive cells, the cells transfected with the construct 1 hr after aggregate formation were used as negative control to determine the gates for cells shifted towards lower EGFP/mCherry ratios after longer time points and thus showed clearance. A figure exemplifying the gating strategy is provided in the Supplementary Information as Supplementary Figure 9.

☒ Tick this box to confirm that a figure exemplifying the gating strategy is provided in the Supplementary Information.
